# A Digital Program (Hope) for People Living With Cancer During the COVID-19 Pandemic: Protocol for a Feasibility Randomized Controlled Trial

**DOI:** 10.2196/24264

**Published:** 2020-12-04

**Authors:** Hayley Wright, Faith Martin, Wendy Clyne, Cain C T Clark, Michael McGillion, Gabriela Matouskova, Andrew Turner

**Affiliations:** 1 Centre for Intelligent Healthcare Faculty of Health and Life Sciences Coventry University Coventry United Kingdom; 2 National Institute for Health Research, Research Design Service South West Peninsula Medical School University of Plymouth Plymouth United Kingdom; 3 School of Nursing McMaster University Hamilton, ON Canada; 4 Hope For The Community, Community Interest Company The Enterprise Hub Coventry United Kingdom

**Keywords:** self-management, survivorship, cancer, feasibility, randomized controlled trial, COVID-19, protocol, digital health, intervention

## Abstract

**Background:**

During the COVID-19 lockdown period in the United Kingdom that began on March 23, 2020, more than a quarter of a million people with cancer reported worsening mental health. Help to Overcome Problems Effectively (Hope) is a self-management program for people with cancer, designed to provide support for distress, unmet needs, and poor psychological health. In light of social distancing during the COVID-19 pandemic, digital delivery of the Hope Programme has become ever more vital for people with cancer. Previous pre-post studies of the digital Hope Programme have found reduced anxiety and depression and improved well-being for people with cancer. However, evaluation of this evidence has been limited by the lack of a control group in these previous studies.

**Objective:**

We now present a protocol for a feasibility randomized controlled trial of the digital Hope Programme for people with cancer during the COVID-19 pandemic. Primary outcomes will be recruitment, dropout, and adherence rates, and estimations of sample and effect size. To detect signals of efficacy, secondary outcomes will be participant mental health and well-being.

**Methods:**

Participants will be recruited by Macmillan Cancer Support (MCS) through their social media networks. The study will employ a feasibility wait-list randomized controlled trial (RCT) design, with people with cancer being randomized to join the digital Hope Programme immediately (intervention group [IG]) or join a 6-week waiting list (wait-list control group [WLCG]) with a 1:1 allocation ratio. Participants will complete digital measures of depression, anxiety, mental well-being, and confidence in managing their own health. Online questionnaires will be administered preprogram and 6 weeks postprogram.

**Results:**

All people who had requested access to the Hope Programme from MCS (N=61) will be invited to participate in the trial. Baseline data collection commenced in April 2020, and the Hope Programme began for the IG in May 2020 and for the WLCG in June 2020. Postprogram data collection was completed by the end of August 2020.

**Conclusions:**

This feasibility study will provide data to inform the design of a future definitive trial. Wider-scale provision of the digital Hope Programme has potential to improve the lives of thousands of people with cancer and reduce the burden on health care providers during these unprecedented times.

**Trial Registration:**

ISRCTN Registry ISRCTN79623250; http://www.isrctn.com/ISRCTN79623250

**International Registered Report Identifier (IRRID):**

DERR1-10.2196/24264

## Introduction

### Background and Rationale

The COVID-19 pandemic has created additional challenges for people with cancer, both in terms of physical and mental health. Many people with cancer are considered at increased risk of serious complications if they were to contract the COVID-19 virus [1]. People with cancer have reported concerns about the further risks to their health from COVID-19, increased anxiety relating to potential cancellation or reduction in treatments and advice from oncology and other medical teams, and significant anxiety and fears of contracting COVID-19 as restrictions in lockdown arrangements are eased [2]. The pandemic has negatively impacted both the cancer health systems and the people using them. Overall, 1 in 5 (20%) people with cancer in the United Kingdom report that they will not feel safe enough to leave their home until an effective treatment or vaccine for COVID-19 becomes widely available [3].

People with cancer are already known to face challenges following primary treatment, including fatigue, pain, sexual problems, issues with cognitive functioning, depression, anxiety, social isolation, and financial issues [4-8]. A significant number of patients with cancer experience long-term negative impacts on their psychological well-being and mental health, including hypervigilance, anxiety, posttraumatic stress, and depression [9-14]; indeed, 2 years postdiagnosis, up to 20% met criteria for major depression and up to 40% met criteria for an anxiety disorder [13,14]. Many of these difficulties are experienced long-term, after regular contact with health care professionals has ceased, which leads to many patients feeling vulnerable and unsupported [5,7,8]. A recent review published before the COVID-19 pandemic came to light highlights the need for urgent research into the longer-term effects of cancer treatment on mental health, as increasing numbers of people live with and beyond cancer [15]. During lockdown, one in four (28%) people with cancer have experienced depression, anxiety, and stress, and one in seven (14%) people with cancer experienced further decline in their physical health (eg, sleep problems, extreme tiredness, and pain) [3]. Macmillan Cancer Support (MCS) have called for the UK government to ensure that holistic physical and mental health support for people with cancer is not forgotten as a result of the COVID-19 pandemic [3].

In the United Kingdom, the National Health Service (NHS) “Long Term Plan” of 2019 [16] places great emphasis on the need for holistic, person-centered care, with greater digital delivery for both NHS services overall and specifically those relating to living well with and beyond cancer. A holistic approach is timely, as recent research shows complex interrelationships between fatigue, fear of cancer recurrence, anxiety, and depression in people with cancer [17,18]. In response to the COVID-19 pandemic, there has also been a rapid and essential growth in the provision of health care digitally, to allow remote care [19,20]. A recent review and meta-analysis showed that digital psychoeducational interventions are effective in significantly reducing depression and fatigue in people with cancer [21]. Many people, including older adults, have become more motivated to use, accepting of, and familiar with digital technologies for health care and social connection [22]. This is essential to meet the needs of people with cancer, as traditional face-to-face support is cancelled and social isolation increases, particularly as in the United Kingdom and many other countries, people with cancer are being advised by the government to isolate or “shield” from others for prolonged periods [23,24].

Around 10 years ago, in response to the shortage of available, tailored self-management support for people with cancer, we codesigned a program together with people with cancer, clinicians, and other experts. The result was a group-based self-management program called the Help to Overcome Problems Effectively Program, known as the “Hope Programme,” for survivors of all types of cancer [25,26], which was originally delivered in-person. The Hope Programme recognizes the common challenges and unmet needs across all types of cancer including fatigue and psychological distress [4-14]. The Hope Programme differs from many other cancer self-management programs due to its roots in positive psychology [27-29] and its focus on hope and gratitude [30] to improve well-being and coping. It has been delivered in person to groups of survivors of all cancers and specifically for survivors of breast cancer, and participants have reported feeling more confident and hopeful and less alone [25,26]. The face-to-face version has been adapted for digital delivery, and initial pretest-posttest evaluation suggested potential effects on anxiety, depression, and positive well-being, with positive user evaluations [31]. A feasibility randomized controlled trial (RCT) study is the next step in the testing of this digital intervention and is required to assess whether participants consent to being randomized, and to test the feasibility of operating a wait-list control group. Owing to COVID-19, in-person service provision has largely been cancelled and this presents the opportunity to conduct the feasibility RCT of the digital intervention. This protocol has been prepared in accordance with SPIRIT (Standard Protocol Items: Recommendations for Interventional Trials) guidelines for clinical trials [32].

### Objectives

The aim of this study is to test the feasibility of a digitally delivered self-management program for people with cancer. This will inform the design of a definitive RCT. Additionally, preliminary assessment of the impact of the Hope Programme, via secondary outcomes, will be used to assess signals of efficacy in a trial context.

The planned primary outcomes (trail feasibility objectives) of the study are to investigate the following:

- Recruitment rates for participation and for randomization
- Retention and follow-up rates as the participants move through the trial
- Adherence rates to study procedures, intervention attendance, and engagement
- Sample size and effect size estimation for a definitive trial
- Progression criteria for a definitive trial

The secondary outcomes are the following:

- Measures of depression, anxiety, confidence to self-manage cancer (patient activation), and mental well-being, as indicated by scores on validated measures

### Trial Design

This study will employ a feasibility, randomized wait-list control group design, to explore the feasibility of a trial of the digital Hope Programme for people with cancer. The intervention is a 6-week digital self-management program. Quantitative monitoring of participant progress through the online program will be undertaken. Participants will be asked to complete standardized measures of depression, anxiety, mental well-being, and confidence in managing their cancer.

## Methods

### Study Settings

This is a digital study and the recruitment, intervention, and data collection will be carried out entirely online. Participants are referred by MCS.

### Eligibility Criteria

Inclusion criteria for the feasibility RCT are the following:

- Diagnosis of any type of cancer, at any stage
- Adult (≥18 years)
- Located in the United Kingdom
- Access to the internet and a device that will allow them to engage with the intervention
- Fluent in English to be able to engage with all the material in the intervention
- Not recruited via the NHS

### Intervention

The Hope Programme will be delivered by Hope for the Community (H4C) Community Interest Company, which is a research social enterprise spinout company from Coventry University [33]. Full details of the digital Hope Programme content are described in Multimedia Appendix 1. All Hope Programme modules have the same structure and format, consisting of quizzes, videos, educational content, activities with homework suggestions, and a module review page. The digital Hope Programme is adapted from the in-person Hope Programme, which was developed in conjunction with cancer survivors and MCS staff. The Hope Programme content comprises text, images, downloadable documents, and links to external websites. The content delivered is configured into interactive activities (eg, quizzes, self-monitoring tools, diaries) that can be used by participants to learn and consolidate the program content. The Hope Programme uses forums and messaging facilities that act as a conduit for communication between participants and facilitators. The Hope Programme is asynchronous, and content is released at set times over the 6 weeks. The Hope Programme is moderated by trained peer facilitators.

### Primary Outcome Measures (Trial Feasibility Objectives)

#### Recruitment Rates

Recruitment rates for participation and randomization will be collected through Qualtrics. All eligible participants identified by MCS will be sent a link to the Qualtrics study survey, so we will calculate recruitment rates from those providing consent and/or completing baseline questionnaires.

#### Retention and Follow-up Rates

Follow-up will be online. Participants who become lost to follow-up will be identified through Qualtrics as those not completing postprogram questionnaires. It is possible that these participants may still complete some or all of the Hope Programme, and so participant retention can be identified separately through engagement with the Hope platform (see below). Participants who explicitly request to be withdrawn from the study will be categorized accordingly, but we will not contact participants to obtain reasons for not completing questionnaires.

#### Adherence Rates

The Hope platform collects user engagement data such as login frequency and duration, which assists the moderators with participant engagement and experience. Participants also have the option of receiving system-generated automatic nudge reminders sent to their email address. We will analyze this user engagement data to generate usage patterns and provide an overview of session attendance and participant engagement.

#### Sample Size and Effect Size Estimation

To inform sample size estimation for a future definitive trial, we will calculate the standard deviations of the continuous secondary outcomes pertaining to depression, anxiety, mental well-being, and participant confidence in managing their cancer. To estimate potential effect sizes for a primary outcome in a future definitive trial (namely, change in scores on key secondary outcome measures from preprogram to postprogram), we will calculate the difference between the mean difference preprogram and postprogram for the intervention and control groups and divide by the pooled standard deviation at baseline [34].

#### Progression Criteria

We will collate the data from all participants in this feasibility RCT to inform progression to a definitive trial, based on the following criteria:

- Recruitment rate >70% of eligible participants consented
- Questionnaire completion rate >70% of participants completing T1 questionnaires
- Program completion rate >50% of participants attending all 6 Hope Programme sessions

### Secondary Outcome Measures

We will administer a sociodemographic and health questionnaire at baseline only, requesting the following personal information from participants: gender, age, ethnicity, marital status, highest level of education, employment and occupation, and some details about their cancer diagnosis and any other medical conditions.

Participants will complete a set of validated questionnaires preprogram and postprogram, to give an indication of changes in depression, anxiety, mental well-being, and confidence to self-manage their cancer across the intervention and control groups. These questionnaires are detailed below.

The Patient Health Questionnaire (PHQ-9) [35] is a 9-item measure that assesses the frequency of depression symptoms (eg, “over the past two weeks, how often have you been bothered by any of the following problems … i) little interest or pleasure in doing things; ii) feeling down, depressed or hopeless; iii) poor appetite or overeating”). Responses to each of the 9 items range from 0 to 3 (0=not at all, 1=several days, 2=more than half the days, 3=nearly every day), leading to a summed score between 0-27, with higher scores indicating greater severity of depression. Scores of ≥10 are presumed to be above the clinical range, and so scores of ≥10 are classed as “cases” of depression. Recovery rates are calculated as those patients who score ≥10 (cases) prior to treatment and <10 posttreatment.

The Generalized Anxiety Disorder scale (GAD-7) [36] is a 7-item scale measuring symptoms of generalized anxiety disorder (eg, “Over the past two weeks, how often have you been bothered by the following problems … i) feeling nervous, anxious or on edge; ii) trouble relaxing; iii) becoming easily annoyed or irritable”). Responses to all 7 items range from 0 to 3 (0=not at all, 1=several days, 2=more than half the days, 3=nearly every day), providing a total score of 0-21, with higher scores indicating greater anxiety. Scores of ≥8 are classed as “cases” of generalized anxiety disorder. Recovery rates are calculated as those patients who score ≥8 (cases) prior to treatment and <8 posttreatment.

The Warwick Edinburgh Mental Wellbeing Scale (WEMWBS) [37] is a scale of 14 positively worded feelings and thoughts, used to assess mental well-being within the adult population. The scale includes measures of positive affect, satisfying interpersonal relationships, and positive functioning (eg, “Below are some statements about feelings and thoughts. Please tick the box that best describes your experience of each over the last two weeks … i) I’ve been feeling optimistic about the future; ii) I’ve been thinking clearly; iii) I’ve been feeling loved”). Participants rate each of the 14 items on a scale of 1 to 5 (1=none of the time, 2=rarely, 3=some of the time, 4=often, 5=all of the time), providing a total positive mental well-being score ranging from 14-70, with higher scores representing greater positive mental well-being. A change of ≥3 is seen as a clinically “meaningful” change [38].

The Patient Activation Measure (PAM) [39] is a validated, licensed tool that has been extensively tested with reviewed findings from a large number of studies. It helps to measure the spectrum of knowledge, skills, and confidence in patients and captures the extent to which people feel engaged and confident in taking care of their condition.

Individuals are asked to complete a short survey and based on their responses, they receive a PAM score (0-100). The resulting score places the individual at one of four levels of activation, each of which reveals insight into a range of health-related characteristics, including behaviors and outcomes. The 4 levels of activation are the following:

Level 1: Individuals tend to be passive and feel overwhelmed by managing their own health. They may not understand their role in the care process.

Level 2: Individuals may lack the knowledge and confidence to manage their health.

Level 3: Individuals appear to be taking action but may still lack the confidence and skill to support their behaviors.

Level 4: Individuals have adopted many of the behaviors needed to support their health but may not be able to maintain them in the face of life stressors.

### Participant Timeline

Participants referred by MCS will be sent an email from H4C, with brief information about the Hope Programme and the feasibility study. The email will contain a link to a Qualtrics survey that contains digital versions of (1) the participant information sheet (PIS), (2) the consent form, and (3) study questionnaires. The PIS and consent form are included in Multimedia Appendix 2. Participants are explicitly informed that they will be randomly assigned to one of two Hope Programmes, starting either in May or in June. Informed consent will be obtained online in accordance with relevant UK legislation (ie, Data Protection Act 2018). The PIS and consent form must be read and agreed to (eg, by checking relevant boxes) before the participant can proceed to the study questionnaires.

After providing informed consent to take part in the study, all participants will be guided through the process of completing the baseline questionnaires (Time 0; hereafter, T0). Upon completion of T0 questionnaires, participants will be randomized via the Qualtrics randomization function, to either attend the Hope Programme starting in May 2020 (intervention group [IG]) or in June 2020 (wait-list control group [WLCG]). Participants are notified which group they have been randomized to at the end of the survey. There will be approximately 30 participants in each group. The IG will then be sent an email link that provides access to the Hope Programme. Those randomized to the WLCG will be informed that they will receive an email link shortly before the start of the Hope Programme in June (ie, approximately 6 weeks later). After 6 weeks (T1), all participants will be emailed a link to the Qualtrics survey containing the secondary outcome questionnaires, and IG participants will also receive a participant debrief at T1. The debrief contains information reminding the participant why the study is being conducted and what will happen to the results, thanking them for their time, and includes sources of additional support if they experienced any distress through completing the questionnaires, such as their own general practitioner or the Samaritans. The WLCG also receive the survey link via email again, with secondary outcome measures and debrief, after they have received the intervention (T2). Table 1 provides a list of all digital study documents presented to participants at each time point (T0, T1, T2). All information and questionnaires at T0, T1, and T2 will be delivered via the Qualtrics survey platform. The Hope Programme will be delivered via the H4C platform.

**Table 1:** Information and questionnaires presented to IG and WLCG participants at each time point (T0, T1, and T2), across the duration of the study.^a^

Study documents	T0	T1		T2
	IG and WLCG	IG	WLCG	WLCG
Participant information sheet	✓			
Informed consent	✓			
Sociodemographic and health questionnaire	✓			
Warwick Edinburgh Mental Wellbeing Scale	✓	✓	✓	✓
Patient Health Questionnaire	✓	✓	✓	✓
Generalized Anxiety Disorder scale	✓	✓	✓	✓
Patient Activation Measure	✓	✓	✓	✓
Poststudy debrief information		✓		✓

^a^T0 refers to the beginning of the study; T1 is 6 weeks later, after the intervention group (IG) has completed the Hope Programme; T2 is an additional 6 weeks later, after the wait-list control group (WLCG) has completed the Hope Programme.

### Sample Size

Participants for this feasibility RCT were drawn from an opportunity sample, referred by MCS, of people with cancer who had expressed an interest in joining the Hope Programme (N=61). As a feasibility study, it was not necessary to conduct sample size calculations to power the study [40]. An arbitrary sample size of n=40 was deemed appropriate for this feasibility study, informed by similar studies in this area [41].

### Recruitment

**Figure 1:**
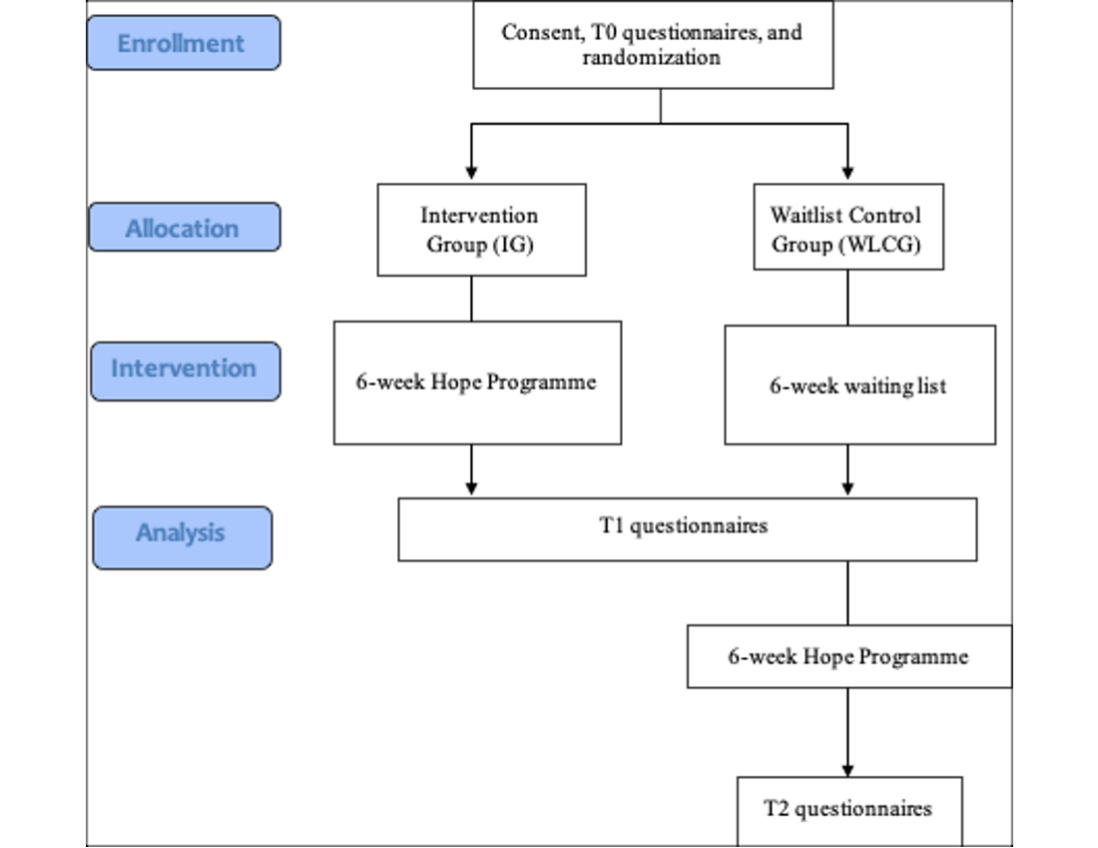
Flow diagram showing participants' route through the study.

We contacted all 61 people with cancer from an opportunity sample of people who expressed interest in attending the in-person MCS Hope Programme and invited them to participate in a trial of the digital program. MCS originally recruited these participants through their social media networks (eg, Macmillan Facebook page and website) and Macmillan Information Centres. Given the urgent need to provide immediate support during the COVID-19 crisis, we did not want to delay the study by seeking National Health Service (NHS) ethics approval to recruit NHS patients. Instead, with University ethics approval in place, we only recruited participants who were recruited via non-NHS sources such as the MCS website and their social media networks. Participant recruitment flow is depicted in Figure 1.

### Assignment of Intervention

#### Allocation Sequence Generation

The participants will be randomly assigned to the IG or WLCG using a 1:1 allocation ratio. Randomization and the allocation sequence will be generated automatically on completion of the online consent form and baseline questionnaires, using the randomization function in Qualtrics Survey Software (Qualtrics). The research team will be unable to influence any aspect of the randomization procedure.

#### Allocation Concealment Mechanism

Participants were informed upon completion of the T0 questionnaires, via a notification in Qualtrics, whether they had been randomized to the IG (in this case, starting in May 2020), or the WLCG (in this case, starting in June 2020). The research team remained unaware of participant allocation until group contact lists were created at the next data collection point (ie, T1).

#### Blinding

It will not be possible to “blind” participants to allocation. Analysis of outcome measures will be conducted blind to participant allocation where possible (eg, IG and WLCG data will be arbitrarily renamed “Group A” and “Group B” for the purpose of analysis).

### Data Collection, Management, and Analysis

#### Data Collection Methods

All data will be collected via online questionnaires administered through Qualtrics. The validated questionnaires (PHQ-9, GAD-7, WEMWBS, and PAM) have been described in the section on Objectives above. Participants will be entered into a prize draw to win a £50 Amazon voucher as an incentive to complete all study questionnaires.

#### Data Management

Each participant in this study will have a unique identifier (ID) generated by Qualtrics at enrollment, and this will be used to link participants’ survey data together at the end of the trial. The user engagement and analytics data collected by the Hope platform will be linked to the survey data by the unique ID. The linked survey and analytics data will be prepared initially in Microsoft Excel (Microsoft Corp) format, and will then be exported to IBM SPSS Statistics 26 (Version 26; IBM Corp), cleaned, and checked for missing values (all data will remain within the shared, password-protected project folder). Once the research team agree that all required data is present and complete, all survey data will be permanently deleted from Qualtrics. The complete anonymized research data files will be stored on the research team’s shared, password-protected project folder located on the university server. To comply with UK regulations (ie, General Data Protection Regulation and the Data Protection Act 2018), the research data will be retained for 3 years after the study has ended and will then be deleted. Only members of the research team will have access to the data files.

#### Statistical Methods

Quantitative data will be analyzed descriptively. Measures of mean and variance, including confidence intervals and standard deviations, and number and percent for categorical variables, will be used to describe the full range of data at baseline and postprogram. An intention-to-treat analysis will be incorporated, where missing data will be rectified using the last observation carried forward [42]. Between-group inferential comparisons will not be performed as the study was not designed to be powered for this analysis, in concordance with the CONSORT (Consolidated Standards of Reporting Trials) extension for pilot and feasibility trials [43]. All analyses will be performed using IBM SPSS Statistics 26.

### Monitoring

#### Data Monitoring

In this small feasibility trial, it was not deemed necessary to employ an independent data monitoring committee. However, participant data was screened at T0, T1, and T2 by the research team to check for indications of suicidal thoughts on the PHQ-9 questionnaire.

#### Harms

Participants who indicate they are feeling suicidal at any point during the study on the PHQ-9 measure will be provided with the contact details of local mental health agencies and Samaritans, and will be encouraged to visit their general practitioner. We will also contact the MCS administrator. At postprogram, all participants’ data will be analyzed to examine if any people have reached a probable clinical level of depression or anxiety where they previously were not at this level. This will be recorded and listed as possible adverse effects of the intervention. These participants will be contacted and encouraged to visit their general practitioner and will be signposted to further sources of support as listed above.

#### Auditing

Auditing of trial conduct will not be necessary. The Hope Programme has been developed and tested in various studies, so the current feasibility trial will focus on recruitment and randomization procedures.

### Ethics and Dissemination

#### Research Ethics Approval

This study was reviewed and approved by the Coventry University Ethics Committee (P106024).

#### Protocol Amendments

Any amendments to the protocol will be submitted to the Coventry University Ethics Committee for review, and any research study activity will be suspended until approval is granted.

#### Consent

Informed consent will be taken online via Qualtrics. Participants will be required to answer “yes” to all consent statements before proceeding to the study questionnaires. If participants answer “no” to any consent statements, they will be directed to the end of the survey and no data will be collected. Consent statements for this study are included in Multimedia Appendix 2.

#### Confidentiality

MCS will send names and email addresses of interested participants to H4C (with the assent of participants), who will email interested participants introductory information and a link to the study website (Qualtrics). At the point of consent, participants will be assigned a unique identifier (ID) through Qualtrics, which will be used to link study data from multiple timepoints (T0, T1, T2). Participant data will be identifiable via this unique ID for the duration of the study. For the prize draw, this unique ID will be linked back to the participant name and email address only for email delivery of the prize (£50 Amazon voucher).

#### Access to Data

HW, FM, CC, and AT will have access to the final data set.

#### Ancillary and Posttrial Care

There is no postprogram follow-up scheduled after the T1 questionnaires for IG, and the T2 questionnaires for WLCG. However, a participant debrief is provided at the end of the study, giving details of where participants can find additional support if they feel they need it.

#### Dissemination Policy

The results of the feasibility RCT will be submitted for publication via the open access route in a relevant journal (eg, Journal of Medical Internet Research), and a lay summary of the findings will be presented in a blog on the H4C website for participants to access. A link to this blog will be emailed to all participants by H4C.

## Results

Recruitment into this feasibility RCT began in April 2020, with all participants completing informed consent and baseline questionnaires before being randomized. Data collection from postprogram questionnaires was completed at the end of August 2020.

## Discussion

### Study Rationale

This feasibility trial is designed to provide evidence about whether it is possible to conduct a definitive trial of a digital self-management program for people with cancer. Digital health care has become more important than ever in the wake of COVID-19, and provision of an acceptable and effective digital self-management program for people with cancer is both timely and necessary [2,19,20].

### Strengths and Limitations

Digital delivery of the Hope Programme has rapidly become essential in light of the COVID-19 pandemic, and the resulting social distancing measures required for those who are clinically vulnerable or required to quarantine. Owing to recent research revealing the number of people with cancer who have experienced declining mental health and increasing fear during the pandemic [3], the Hope Programme is well placed to deliver timely and much-needed support for people with cancer, particularly while treatments and contact with health care teams are reduced [2].

It is noteworthy that there is not always a linear relationship between time spent in a digital intervention, the number of sessions completed, and participant outcomes [44]. For example, it is possible that the participants who access only a couple of sessions may be “e-attainers” (as described in [45]). These participants may achieve what they need from the program, such as practicing gratitude, or gaining reassurance that their challenges are shared by others [46]. In our comprehensive statistical analyses, we plan to use a combination of data from our usability measures and the user engagement data extracted from the platform to develop a more thorough understanding of engagement and attrition, which can then be used to inform the content and design of future versions of the Hope Programme.

A challenge for many digital interventions is completion of postprogram questionnaires. Achieving an acceptable postprogram questionnaire completion rate is a key criterion for progression to a definitive trial. In this feasibility trial, we have included entry to a prize draw for those that complete the postprogram questionnaires. This will enable us to determine whether this is sufficient to secure an acceptable postprogram completion rate in this population. Exploring the feasibility of achieving acceptable completion rates for questionnaires is particularly pertinent with a wait-list control group design. There is some evidence that wait-list controls for studies of psychological interventions may overestimate intervention effects relative to other forms of control group [47]. Wait-list control participants are hypothesized to delay taking action while they are waiting relative to no active treatment control participants [48]. Testing the feasibility of this approach will enable us to establish whether this is an acceptable trial design and an ethical approach to RCTs for people with cancer in these unprecedented times.

## Appendix

Multimedia Appendix 1 Hope Programme development and content.

Multimedia Appendix 2 Informed consent materials.

## Abbreviations

CONSORT: Consolidated Standards of Reporting Trials
GAD-7: Generalized Anxiety Disorder scale
H4C: Hope for The Community
MCS: Macmillan Cancer Support
NHS: National Health Service
PAM: Patient Activation Measure
PHQ-9: Patient Health Questionnaire
PIS: participant information sheet
SPIRIT: Standard Protocol Items: Recommendations for Interventional Trials
WEMWBS: Warwick Edinburgh Mental Wellbeing Scale
