# Supplementary material for: A Digital Program (Hope) for People Living With Cancer During the COVID-19 Pandemic: Protocol for a Feasibility Randomized Controlled Trial
Source: JMIR Res Protoc. 2020 Dec 4;9(12):e24264. doi: 10.2196/24264 (PMC7721632; doi:10.2196/24264)
Supplement: Multimedia Appendix 1 [file resprot_v9i12e24264_app1.docx]

## Appendix 1: Hope Programme development and content.

### Overview

In response to the shortage of available, tailored self-management support programmes for cancer survivors, we worked with cancer survivors, clinicians and other experts to develop a group-based, face-to-face self-management programme: Help to Overcome Problems Effectively, known as the “HOPE” Programme, for survivors of all types of cancer.

The HOPE programme aims to enhance well-being by fostering positive emotions and stimulate positive functioning. A parallel goal is to reduce depressive symptoms. The HOPE programme is based on principles derived from positive psychology and focuses on positive experiences, strengths, and personal competencies rather than mental health problems such as anxiety and depression. It incorporates evidence-based exercises based on positive psychology, in addition to elements stemming from mindfulness, cognitive behavioural therapy and problem-solving therapy.

### Underlying principles

The HOPE programme is distinct from other cancer self-management programmes due to its roots in positive psychology [1-3], and its unique focus on hope and gratitude to create an upward spiral of positivity [4] to improve wellbeing and coping. Fredrickson [5] shows that increasing positive emotions broadens attention, thinking and action, which enables people to develop more creative thought and action pathways (e.g. expanding coping skills), and thus develop crucial personal and social resources for self-management.

Hope theory [6,7] is similar to self-efficacy theory, but whereas the latter focusses on specific goals and behaviours, hope theory recognises enduring cross-situational goals and behaviours, and as such is better suited to the complexity of managing the diverse impact of long term conditions. Goals are fundamental in hope theory, which encompasses a cognitive set that is based on both agency (goal-directed determination) and pathways (planning ways of achieving goals).

Gratitude has been shown to improve psychological wellbeing and increase positive emotions [8,9] with some interventions showing that increasing gratitude is linked to improvements in depression [10]. A gratitude activity is a weekly feature in the HOPE programme, and is designed to increase participants’ positive emotions. The HOPE programme also includes other evidence-based CBT and positive psychological activities such as identifying personal strengths, scheduling pleasant activities, mindfulness, relaxation training and reviewing successes.

Group curative factors of instilling hope, universality, and altruism [11] are embedded within the HOPE programme content, where participants observe their peers overcoming challenges and achieving goals (‘instillation of hope’), share experiences (‘universality’) and provide informational and emotional support for each other (‘altruism’). Thus, drawing on the principles of positive psychology, hope theory and gratitude, and embedding group curative factors, the HOPE programme provides participants with a novel toolkit to develop skills and resources to improve their wellbeing and quality of life, during and beyond treatment.

### Digital programme development

The HOPE Programme recognises the common challenges and unmet needs across all types of cancer including fatigue, fear of recurrence and psychological distress [12-22]. The HOPE Programme was co-designed with service users and stakeholders from one of the UK’s leading cancer charities, Macmillan Cancer Support [23,24].

The HOPE Programme has been taxonomised using the taxonomy of self-management support [54]. The adaptation to the digital HOPE programme was undertaken in consultation with people who had attended, delivered and commissioned the delivery of the face-to-face HOPE programme. A user-centred, iterative approach was undertaken [55], as detailed below. A set of design requirements and a design brief were drawn up in consultation with end-users and stakeholders. It was specified that the digital version of HOPE should replicate the process and content of the group-based HOPE course, to ensure that marketing and recruiting of both versions of the course would be consistent, and that cross training of face to face facilitators to deliver the digital HOPE programme would be kept to a minimum. The initial digital version of HOPE went through a number of iterative testing sessions, with improvements made to usability after each iteration. It was intended through these iterations to develop a system that was useable and accepted by the intended user group to increase the likelihood of uptake and continued usage, and ensure the technology did not prove a barrier to engagement and participation.

Iteration 1: We carried out usability testing of the digital HOPE programme with stakeholders including MCS staff, trainers and cancer survivors. A link to the course was circulated to past participants and facilitators of the face to face programme. They were asked to work through the online material and provide feedback to specific questions via email. It was aimed to explore whether the core components of the digital HOPE programme were integrated effectively.

Iteration 2: The digital HOPE programme was then reviewed by a wider audience and demonstrated to delegates at the National Cancer Voices conference in November 2013. This round of feedback focused on the acceptability of the translation of face to face course features into an online format.

Iteration 3: MCS and Coventry University researchers held a workshop with the web design team and experienced HOPE facilitators, to collect feedback on the digital HOPE programme design, usability and content. User and facilitator feedback led to further revisions to improve usability and course experience.

Iteration 4: The final iterative feedback was undertaken as part of the digital HOPE programme evaluation, and five cohorts reviewed the system whilst enrolled in the programme.

### HOPE programme content

The HOPE programme content comprises text, images, downloadable documents, links to external websites, for example activities and media relating to cancer-related fatigue and developing character strengths. The content delivered is configured into interactive activities (e.g. quizzes, self-monitoring tools, diaries) that can be used by participants to learn and consolidate programme content. The HOPE programme uses forums and messaging facilities that act as a conduit for communication between participants and facilitators.

The digital HOPE programme content is released weekly over six weeks, and is an asynchronous programme (not requiring real time attendance). On the same day each week, new content is released, whilst previous content remains available. Participants are encouraged to log on for around 2.5 hours per week and use a range of behaviour change techniques including: weekly goal setting, action planning and self-monitoring. Peer support and interaction is facilitated through social networking tools and shared interactive activities where all participants’ comments appear. Weekly topics and activities are provided in Table 1.

Table 1. Weekly topics, content, exercises and activities included in the six week iHOPE programme

| Session | Examples of content | Examples of self-management tools, exercises and activities |
| --- | --- | --- |
|  |  |  |
| Week 1: Introduction/Instilling hope | Aims of the programme.  User guide to navigating the platform and setting up a profile.  Introduction to self-management.  The benefits of positive emotions.  Video: positive emotions for a flourishing life.  The power of gratitude.  Personalised goal setting.  Video: How to set achievable goals.  Forum topic: Reasons for joining the programme.  Further resources & links (e.g. videos, podcasts, websites) to gratitude, positivity, goal setting. | Interactive gratitude diary; SMARTER^a^ goal setting.  Assessment: Positivity ratio test and positive & negative emotions test. |
| Week 2: Stress management | Understanding stress. Managing stress.  Videos: How to manage stress; How to make stress your friend.  Coping with unhelpful thinking patterns.  Mindfulness for stress management and meditation.  Self-compassion and acceptance.  Video: How to be kind to yourself.  Forum topic: How do you deal with cancer-related stress?  Further resources & links (e.g. videos, podcasts, websites) to self-compassion, mindfulness, stress management. | Interactive gratitude diary; SMARTER goal setting & goal feedback.  Guided relaxation and meditation exercise (podcasts).  How to cope with unhelpful thoughts (worksheet). |
| Week 3: Managing fatigue | Understanding the ‘boom and bust’ cycle.  Using the 3 Ps (prioritising planning, pacing,) for managing fatigue.  Video: Tips for managing fatigue.  Sleeping better.  Podcast: Tips to improve sleep.  Forum topic: Coping with fatigue.  Further resources & links (e.g. videos, podcasts, websites) to sleeping better. | Interactive gratitude diary; SMARTER goal setting & goal feedback.  Fatigue and pacing diaries  (worksheets).  Quiz: What are the main challenges faced by cancer survivors? |
| Week 4: Body image and communication | Body image.  Video: Body image and cancer.  Sexuality and intimacy.  Video: Cancer as a passport to emotional intimacy. Communication skills and tips for talking with healthcare team and family.  Forum topic: Experiences of coping with body changes & experiences of communicating with healthcare team.  Further resources & links (e.g. videos, podcasts, websites) to sexuality, intimacy and relationships. | Interactive gratitude diary; SMARTER goal setting & goal feedback. |
| Week 5: Physical activity and fear of recurrence | Coping with fear of recurrence.  Videos: Moving forward while being worried about cancer returning; Regrets of the dying.  Hopes and dreams for the future.  Video: Before I die project.  The benefits of physical activity.  Video: Tips for becoming and staying active.  Forum topic: Concerns about cancer coming back.  Further resources & links (e.g. videos, podcasts, websites) to managing concerns about cancer coming back and getting more active. | Interactive gratitude diary; SMARTER goal setting & goal feedback. |
| Week 6: Character strengths and happiness | Understanding how using your strengths can lead to a more fulfilling life.  Video: The science of character strengths.  Tips for authentic happiness.  Managing setbacks and keeping going.  Forum topic: Learning from the programme;  Further resources & links (e.g. videos, podcasts, websites) to Macmillan Cancer Support online communities and happiness resources. | Interactive gratitude diary; SMARTER goal setting & goal feedback.  Assessment: Positivity ratio test and positive & negative emotions test and character strengths.  Quiz: What contributes to happiness? |

^a^SMARTER: SMARTER is an acronym used by many organizations for goal setting, and stands for: Specific, Measurable, Achievable, Relevant, Time-bound, Enjoyable, Reward.

Goal setting and gratitude activities are recurring weekly features and participants are encouraged to post a goal or something they may feel grateful for on the ‘online walls’ for everyone to see and comment on. Each week, a number of questions are set as discussion topics which are featured in the weekly forums. These questions are related to the programme content for each week. There is also a weekly forum (Hope Lounge) where participants can start a discussion about either their experience of living with and beyond cancer or a non-cancer related topic.

The digital HOPE programme is moderated by two trained peer facilitators who themselves are affected by cancer in some way. The facilitators have received training from MCS and follow a delivery protocol. The facilitator’s role is to offer encouragement to participants, stimulate discussion in social networking forums by inviting participants to respond with comments to specific questions, or respond to questions/comments posted by participants. Facilitators also monitor the daily social networking posts for safety and report any technical problems to the research team. Facilitators spent two hours each per session supporting the participants.

### References

1. Seligman, M.E.P. (1991). Learned Optimism: How to Change Your Mind and Your Life. New York, NY: Pocket Books.

2. Seligman, M.E.P. (2002). Authentic Happiness: Using the New Positive Psychology to Realize Your Potential for Lasting Fulfillment. New York, NY: Free Press.

3. Linley PA, Joseph S, editors. Positive psychology in practice. New York: John Wiley & Sons; 2004.

4. Tugade MM, Fredrickson BL, Feldman BL. Psychological Resilience and Positive Emotional Granularity: Examining the Benefits of Positive Emotions on Coping and Health. Journal of Personality. 2004;72(6):1161-90. PMID: [15509280](https://www.ncbi.nlm.nih.gov/pubmed/15509280)

5. Fredrickson, B. L. (2001) The role of positive emotions in positive psychology: The broaden-and-build theory of positive emotions. *American psychologist*, 56(3), 218-226. PMID: [11315248](https://www.ncbi.nlm.nih.gov/pubmed/11315248)

6. Snyder, C. R., Harris, C., Anderson, J. R., Holleran, S. A., Irving, L. M., Sigmon, S. & Harney, P. (1991) The will and the ways: development and validation of an individual-differences measure of hope. *Journal of personality and social psychology*, 60(4), 570-586. PMID: 2037968

7. Snyder CR. Hypothesis: there is hope. In: Snyder CR, editor. Handbook of hope: theory, measures and application. New York: Academic Press; 2000. p. 3–18.

8. McCullough, M. E., Tsang, J.-A., & Emmons, R. A. (2004). Gratitude in Intermediate Affective Terrain: Links of Grateful Moods to Individual Differences and Daily Emotional Experience. Journal of Personality and Social Psychology, 86(2), 295–309. PMID: 14769085

9. Wood AM, Maltby J, Gillett R, et al. The role of gratitude in the development of social support, stress, and depression: two longitudinal studies. J Res Person. 2008; 42: 854–871. DOI: [10.1016/j.jrp.2007.11.003](https://www.researchgate.net/deref/http%3A%2F%2Fdx.doi.org%2F10.1016%2Fj.jrp.2007.11.003?_sg%5B0%5D=k9A60mryd9Z-j9mR9sxMyO50hhTRRhynewc2l6JETC8feTza6CJQxlAGq2sZdg_k9yq3BHjNyRMGEjvdIzM5i9fS0g.HINamqGY7StCaJ6NC-j87w11ws1WYJBHH4FzxnPhaBO2NQBNPqouSLfUqvgh2fyol-1cS6r1cmxcFez75uLhHQ)

10. Bono G, McCullough ME. Positive responses to benefit and harm: bringing forgiveness and gratitude into cognitive psychotherapy. J Cogn Psychother. 2006; 20: 2; 147-158. DOI: 10.1891/jcop.20.2.147

11. Yalom I. Theory and Practice of Group Psychotherapy. 5th ed. New York: Basic Books; 2005.

12. Armes, J., et al., Patients' supportive care needs beyond the end of cancer treatment: a prospective, longitudinal survey. Journal of Clinical Oncology; 2009. 27(36): 6172-6179. PMID: 19884548

13. Foster, C. and D. Fenlon, Recovery and self-management support following primary cancer treatment. British Journal of Cancer; 2011. 105(S1): S21. PMID: 22048029

14. Elliott, J., et al., The health and well-being of cancer survivors in the UK: findings from a population-based survey. British Journal of Cancer; 2011. 105(S1): S11. PMID: 22048028

15. Cella, D., et al., Cancer-related fatigue: prevalence of proposed diagnostic criteria in a United States sample of cancer survivors. Journal of Clinical Oncology; 2001. 19(14): 3385-3391. PMID: 11454886

16. Hofman, M., et al., Cancer-related fatigue: the scale of the problem. The Oncologist; 2007. 12(Supplement 1): 4-10. PMID: 17573451

17. Simard, S., Thewes, B., Humphris, G., Dixon, M., Hayden, C., Mireskandari, S., Ozakinc, G. (2013). Fear of cancer recurrence in adult cancer survivors: a systematic review of quantitative studies. Journal of Cancer Survivorship, 7, 300. PMID: 23475398

18. Simard, S., J. Savard, and H. Ivers, Fear of cancer recurrence: specific profiles and nature of intrusive thoughts. Journal of Cancer Survivorship; 2010. 4(4): 361-71. PMID: 20617394

19. Koch, L., et al., Fear of recurrence in long‐term breast cancer survivors—still an issue. Results on prevalence, determinants, and the association with quality of life and depression from the Cancer Survivorship—a multi‐regional population‐based study. Psycho-Oncology; 2014. 23(5): 547-554. PMID: 24293081

20. Koch, L., et al., Fear of recurrence and disease progression in long‐term (≥5 years) cancer survivors—a systematic review of quantitative studies. Psycho-Oncology; 2013. 22(1): 1-11. PMID: 22232030

21. Mitchell, A.J., et al., Depression and anxiety in long-term cancer survivors compared with spouses and healthy controls: a systematic review and meta-analysis. The Lancet Oncology; 2013. 14(8): 721-732. PMID: 23759376

22. Inhestern, L., et al., Anxiety and depression in working-age cancer survivors: a register-based study. BMC Cancer; 2017. 17: 347. PMID: 28526007

23. Turner, A., et al., HOPE: A Positive Psychological Group Self-Management Support Programme for Cancer Survivors. Psycho-oncology; 2012. 21(S2): 16-16. DOI: 10.1111/j.1099-1611.2012.03051.x

24. Martin, F., et al., Systematic Development of a General Self-Management Intervention for Survivors of Cancer; 2010, University of Stirling, Scotland May 11th - 13th 2010: Poster presented at International Conference on Support for Self Management of Health.
