# Supplementary material for: A Digital Program (Hope) for People Living With Cancer During the COVID-19 Pandemic: Protocol for a Feasibility Randomized Controlled Trial
Source: JMIR Res Protoc. 2020 Dec 4;9(12):e24264. doi: 10.2196/24264 (PMC7721632; doi:10.2196/24264)
Supplement: Multimedia Appendix 2 [file resprot_v9i12e24264_app2.docx]

## Appendix 2: Informed consent materials.

### Participant Information Sheet

**A feasibility randomised controlled trial of the digital Hope Programme for people living with cancer during COVID-19**

This information sheet is designed to give you more information about the project. It is for you to keep for your own use. Please read this information carefully before deciding if you would like to participate in the study. Please ask any questions that you may have about the project.

**It is important to note that taking part in this study is not a substitution or replacement for psychological therapy or medical treatment, it is designed to be used alongside existing treatment options. Please contact your GP or another healthcare professional if you feel you would benefit from psychological therapy.**

**Information about the project:**

**What is the purpose of the study?**

We want to test whether a six-week online self-management programme, called the Hope Programme, is acceptable to people with cancer and whether it can help them self-manage better during the COVID-19 crisis.

**Why have I been chosen?**

You have been invited to take part because you have chosen to register for the Hope Programme self-management course. Everyone who registers will be invited to take part.

**Do I have to take part?**

No. It is up to you. If you decide not to take part in the study, you can still have access to the Hope Programme. If you do decide to take part, please keep this Information Sheet and complete the Informed Consent Form to show that you understand your rights in relation to the research. Even if you consent, you are free to withdraw from the study or any parts of it at any time, up until 31^st^ July 2023.

Please note that your data may be used anonymously in research outputs (e.g. journal articles, conference papers, theses and reports) prior to this date and so you are advised to contact the university at the earliest opportunity should you wish to withdraw from the study.

You do not need to give a reason for withdrawing from the study and neither participation nor withdrawal will have any impact on the care you receive.

**What do I have to do?**

If you decide that you would like to take part, we will ask you to complete an online consent form and questionnaire. You will be asked a number of questions about your name, email address, gender, age, postcode, occupation, highest level of education and some details about your quality of life, emotional health and confidence to self-management. Your name and email address will not be stored alongside your responses. We are only collecting postcode data to help us to understand whether we reach a range of people in our study. As there is a possibility that your postcode, in combination with your personal data, could be used to identify, we will calculate a deprivation score immediately when we download your data and then delete your postcode straight away. For more information please see the section on **‘How would you process my data?’** below.

The questionnaires should take around 20 minutes to complete. Macmillan Cancer Support are running two Hope Programmes; one in May 2020 and another in June 2020. We are recruiting 60-70 people for each group. The data collected from each group will be equally important and the researchers have no control over who gets assigned to which group. Once you have completed the pre-course questionnaires at enrolment, an online tool will randomly assign people to each group.

Once you have completed the pre-course questionnaires, you will be randomised to either start the Hope Programme straight away in May, or in 6 weeks’ time in June. If you start the course in June, you will be asked to complete the same questionnaires again before you begin the course. So, the questionnaires will be completed prior to randomisation onto the May or June course and again at the end of the programme (i.e. post-course) by all participants, but those on the June course will be asked to complete an additional set prior to starting the course.

When you join the Hope Programme you will log in over a period of six weeks, to access weekly course materials and interact with other users. You will need to have access to the internet and an internet-enabled device (e.g. phone, tablet, desktop computer or laptop) so you can access the course and complete the study surveys.

**What are the risks associated with this project?**

There are no direct risks of taking part in this study. Whilst we do not anticipate the questionnaires will cause you any distress, some of the questions will ask about your health, which may touch on topics that you may find sensitive. However, you do not have to answer any questions that you are uncomfortable with.

If you do experience any distress from completing the questionnaires, please contact your GP, or the Samaritans on 116 123 (24 hour telephone helpline) or [jo@samaritans.org](mailto:jo@samaritans.org) (you will receive an email response within 24 hours).

**What are the benefits of taking part?**

The online Hope Programme has been shown to be helpful for a small group of people living with cancer in a study which did not have a control group. You may find the course helpful and/or you may have suggestions for how it could be improved before it is put into a full scale clinical trial. We will enter you into a prize draw for Amazon Gift vouchers for £50 for completion of two questionnaires.

**How would you process my data?**

You are currently on the research website Qualtrics. Qualtrics is an application for creating and hosting online questionnaires. It has been assessed and approved by Coventry University’s Information Protection Unit.

If you decide to take part in this study, you will be asked to register on Qualtrics. This will involve providing your name and email address. We will use this information to contact you about the research study. Researchers from Coventry University and regulatory organisations may look at your research records to check the accuracy of the research study. The only people at Coventry University who will have access to information that identifies you will be researchers who will use this to contact you or to audit the data collection process. The people who analyse the information will not be able to identify you and will not be able to find out your name or contact details.

We will collect standard internet and website login information and details of patterns about how frequently and for how long you access the Hope Programme. This information will help us improve your course experience. We will collect data on how the group uses the Hope Programme. For example, how many times the group visits specific pages and the date and time of those visits. This will allow us to find out which parts of the Hope Programme are more popular and useful. This information will be collected via a software tool such as Matomo**,** which is a Coventry University hosted web analytics software.

Your data will be processed in accordance with the General Data Protection Regulation 2016 (GDPR). Coventry University is the sponsor for this study. We will be using information from you in order to undertake this study and will act as the data controller for this study. This means that we are responsible for looking after your information and using it properly.

Your rights to access, change or move your information are limited, as we need to manage your information in specific ways in order for the research to be reliable and accurate. If you withdraw from the study, we will keep the information about you that we have already obtained. To safeguard your rights, we will use the minimum personally-identifiable information possible. In accordance with the Coventry University data retention policy, we will destroy all study data on 31^st^ July 2023.

**Data protection & confidentiality:**

The questionnaires you complete will be seen only by the research team. Any identifiable electronic data will be stored on encrypted devices and central servers serviced by Coventry University. All the information will be kept strictly confidential and held in accordance with the principles of the Data Protection Act 1998 and 2003, and General Data Protection Regulation 2016 (GDPR).

No individuals will be identified in any reports or publications. All participant data will be assigned an ID code. This ID code will just be used to link your three or four questionnaires together so that we can compare your responses, and will not be identifiable to you. This study will comply with the Data Protection Act 1998 and 2003, and General Data Protection Regulation 2016 (GDPR) and your participation will be kept confidential. Only members of the research team will have access to the data.

The consent form that you sign digitally will be saved on a password protected computer (separate to your questionnaire responses).

**What if things go wrong? Who to complain to:**

You can contact the lead researcher (Professor Andy Turner) at any time during the project if you are not happy. If you are still not happy, you can contact the Research Ethics Lead for the Faculty of Health and Life Sciences at Coventry University (Dr Helen Breadmore). Contact details are provided below.

**What will happen with the results of the study?**

The anonymised findings will be used to improve the Hope Programme courses to help people manage their long-term conditions.

**Who has reviewed this study?**

Coventry University and NHS Ethics Committee has reviewed and approved this study.

**Further information/Key contact details**

**Lead Researcher**

Professor Andy Turner

Faculty of Health and Life Sciences (HLS)

Coventry University

Priory Street

Coventry CV1 5FB

a.turner@coventry.ac.uk

07392 096520

**In case of complaint:**

Dr Helen Breadmore

Research Ethics Lead for HLS

Coventry University

Priory Street

Coventry CV1 5FB

helen.breadmore@coventry.ac.uk

07974 984020

### Informed Consent Statements

[The consent form was presented digitally online. All statements must be agreed before the participant could commence the study questionnaires]

1. I confirm that I have read and understood the Information Sheet for the Hope study, and that I have had the opportunity to ask any questions
2. I understand that you will be collecting the following data from me: basic sociodemographic information e.g. postcode, age, gender, basic information about my condition, mental health and wellbeing measures (these will be collected using separate measures)
3. I understand that all the information I provide will be treated in confidence
4. I understand that my participation is voluntary and that I am free to change my mind and withdraw at any time, without giving a reason, and that my data can be deleted up until (31/07/2023) by emailing (a.turner@coventry.ac.uk)
5. I understand that the data from the Hope study may be used in an anonymised form for scientific publications and presentations
6. I agree to take part in the study
